# Hybrid revisional surgery: biliary limb distalization plus endoscopic transoral outlet reduction (eTOR)

**DOI:** 10.1093/jscr/rjac177

**Published:** 2022-05-14

**Authors:** Guillermo Borjas, Nestor Sánchez, Ali Urdaneta, Andres Maldonado, Eduardo Ramos, Edward Fumero, Jose DiGiorgio

**Affiliations:** Unidad Internacional de Cirugía Bariátrica y Metabólica – Clínica “La Sagrada Familia”, Maracaibo, Venezuela; Grupo Medico Santa Paula, Caracas, Venezuela; Unidad Internacional de Cirugía Bariátrica y Metabólica – Clínica “La Sagrada Familia”, Maracaibo, Venezuela; Grupo Medico Santa Paula, Caracas, Venezuela; Unidad Internacional de Cirugía Bariátrica y Metabólica – Clínica “La Sagrada Familia”, Maracaibo, Venezuela; Grupo Medico Santa Paula, Caracas, Venezuela; Unidad Internacional de Cirugía Bariátrica y Metabólica – Clínica “La Sagrada Familia”, Maracaibo, Venezuela; Grupo Medico Santa Paula, Caracas, Venezuela; Unidad Internacional de Cirugía Bariátrica y Metabólica – Clínica “La Sagrada Familia”, Maracaibo, Venezuela; Grupo Medico Santa Paula, Caracas, Venezuela; Unidad Internacional de Cirugía Bariátrica y Metabólica – Clínica “La Sagrada Familia”, Maracaibo, Venezuela; Grupo Medico Santa Paula, Caracas, Venezuela; Unidad Internacional de Cirugía Bariátrica y Metabólica – Clínica “La Sagrada Familia”, Maracaibo, Venezuela; Grupo Medico Santa Paula, Caracas, Venezuela

## Abstract

Roux-en-Y gastric bypass (RYGB) is one of the best procedures for the treatment of obesity and associated comorbidities. However, the percent of revisional procedures after a gastric bypass by weight regain has been increased, therefore several surgical options are available for the treatment of weight regain. In this case report, we combined a biliary limb distalization with endoscopic transoral outlet reduction (eTOR). The purpose of this case report is to expose the viability to perform combined procedures such as the distalization of the biliopancreatic limb plus eTOR increasing malabsorptive and restrictive components that would represent a secure and efficient weight loss in our patient. We could demonstrate the technical feasibility of the combination of both procedures to increase the restrictive and malabsorptive components at the same time with a low-risk range.

## INTRODUCTION

Weight regain is defined as a gain of weight of >15% of maximum weight loss; currently is a common problem in the multiple techniques of bariatric surgery. In Roux-en-Y gastric bypass (RYGB), 20–50% percent of patients experience significant weight regain in 5–10 years after surgery and weight regain can occur as early as 1 year after RYGB [1, 2].

Failure can be defined as inadequate weight loss or weight regain. The Adelaide study group proposed using an excess weight loss (EWL) >50%, as previously described by Reinhold [3], whereas Fobi *et al*. utilize the term ‘failure’ for EWL < 40% [4]. Failure is usually multifactorial and can be attributed to the surgeon (technical issues such as a large pouch in RYGB), to the patient (behavioral and dietary habits) and to the disease process itself (bariatric surgery ‘resistance’).

There are multiple treatments in revisional surgery; including surgical or endoscopic procedures. Classically these techniques are not performed in combination but based on the rationale that by applying each one at the same time we can increase the malabsorptive and restrictive component in a single step making synergy in the mechanism of weight loss. Our objective with this case report is to present a new technique of revisional surgery, which we named hybrid surgery and to demonstrate its technical feasibility and safety.

## CASE REPORT

A 52-year-old male with hypertension and controlled atrial fibrillation had a laparoscopic RYGB in 2011 when he had a weight of 173 kg, height of 1.82 m and body mass index (BMI) of 52.52 kg/m^2^. The minimum weight reached by the patient was 112 kg, which is equivalent to 62% of the EWL. The patient consulted in the second semester of 2020 to our facility for weight regain, his weight reached 139.7 kg, BMI of 42.17 kg/m^2^ that corresponds to weight regain of 44.67%. In the multidisciplinary evaluation, we performed laboratory work-up with normal parameters and pre-operative endoscopy that reports a stoma size of 30-mm approximately.

**Figure 1:**
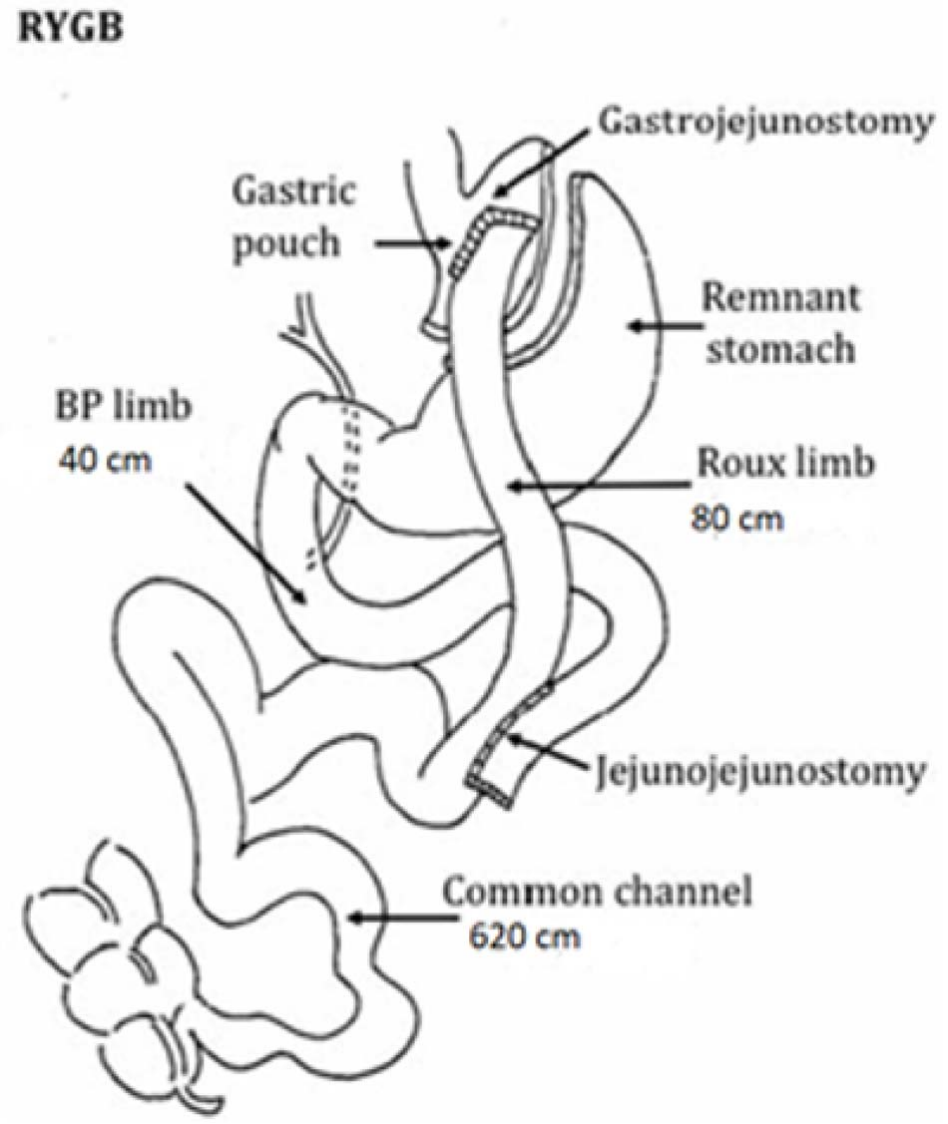
Findings of primary surgery.

**Figure 2:**
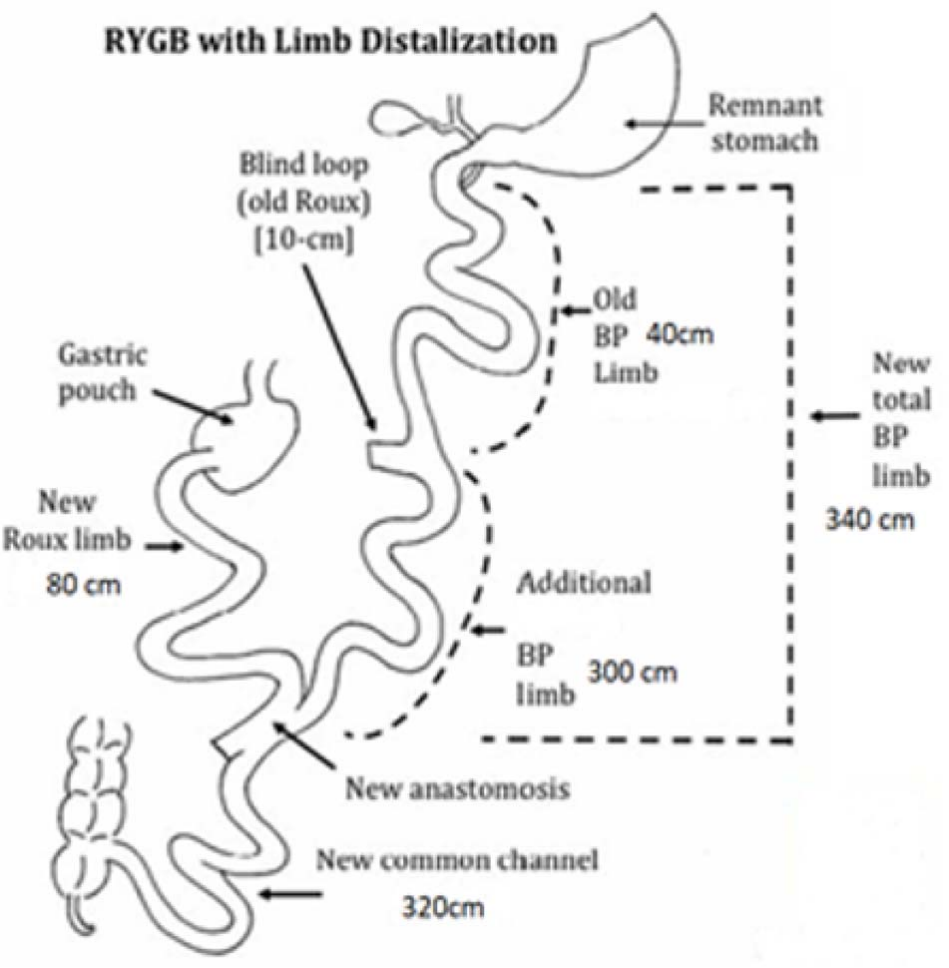
Measures after limb distalization. (Reference images were taken from: Surve, A, Cottam, D. A step-by-step surgical technique video of revision of Roux-en-Y gastric bypass with limb distalization. *OBES Surg*  **31**:464–466. Those images were modified in order to explain our procedure).

**Figure 3:**
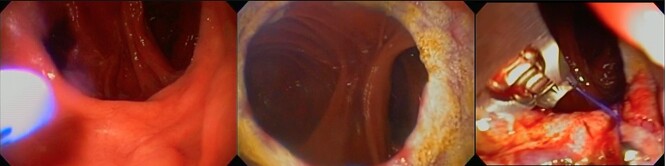
eTOR procedure: (**a**) view of the GJ anastomosis, (**b**) APC therapy and (**c**) eTOR.

Revisional bariatric surgery is performed with the patient in french position by a bariatric surgeon with many years of experience. During the bowel exploration, we found an alimentary limb of 80 cm, a biliopancreatic (BP) limb of 40 cm and a common channel of 625 cm (Fig. 1). A distalization of the BP limb was performed, using a blue load to resect the intestinal segment of the alimentary limb proximal to the jejunojejunal anastomosis. Posteriorly, we began to measure the common channel to create the new jejunojejunal anastomosis at 320 cm of the ileocecal valve distalizing the BP limb to 340 cm and shortening the common channel to 320 cm (Fig. 2). Afterward, assessing via endoscopy a gastric pouch of 5 cm is measured with a large non-functional limb (jejunal cane) and a stoma size of 30 mm, it is decided to perform eTOR procedure using the OverStitch™ platform, reducing the stoma size from 30 to 10 mm approximately (Fig. 3). Both procedures last 200 min without complications. The patient was discharged after 48 h under nutritional indications. Ten months after surgery the patient has lost 31.7 kg with a BMI of 32.60 kg/m^2^ without eventualities, taking nutritional supplements.

## DISCUSSION

Because of the security and efficacy of bariatric surgery to treat obesity the number of procedures performed worldwide has increased importantly. Is not a surprise that the revisional procedures for weight regain have increased as well. There are several options to treat weight regain, being conscious of the multifactorial reasons that aid this failure in weight loss. This is why we present this innovative hybrid revisional surgery technique as a possible alternative.

Studies have demonstrated that a dilated gastrojejunal stoma after RYGB is a strong independent risk factor associated with weight regain and the diameter of the gastrojejunal stoma demonstrates a positive linear correlation with the amount of weight regained [5]. The endoscopic transoral outlet reduction (eTOR) procedure has emerged as the most widely used technique worldwide, because of its safety and effectiveness for the treatment of weight regain after RYGB. Vargas *et al.* demonstrated the efficacy of the treatment of weight regain in RYGB performing eTOR procedure with an average of stoma diameter 28 ± 4.74 mm in 130 patients, the average of weight loss reported at 6, 12 and 18 months was 9.31 ± 6.7 kg, 7.75 ± 8.4 kg and 8 ± 8.8 kg, respectively [6].

On the other hand, distalization of the BP limb as an alternative for RYGB revisional surgery has shown equally good results in terms of weight loss. The role of length of the BP limb in super-obese patients is important and has been demonstrated that a 2-m BP limb has effective results for weight loss, also the importance of not shortening the common channel beyond 200 cm to avoid nutritional complications [7].

BP limb distalization is an acceptable option and its use has been increasing along the year for surgeons worldwide for revisional surgery after RYGB for the exceptional results in weight loss, however, there is a high risk of malnutrition, protein and vitamins deficit if the common channel is shortened <300 cm [4, 8, 9]. This technique, besides having effective results in weight loss, also has good results in diabetes and hypertension remission with 69.9 and 59.8%, respectively [10].

In our experience, we did another case the last year, which prove the feasibility in the use of laparoscopic BP limb distalization and endoscopic transjejunal APC pouch therapy in a revisional surgery for RYGB, obtaining good results in %EWL in a patient with 49.7% of weight regain, no transoperative complication was reported [11].

It seems to be a reliable and feasible combination of both procedures to increase the restrictive and the malabsorptive components at the same time. To date to our knowledge, there are no studies or protocols published about this combination, however, we share our experience with this case report and aim to other surgeons to further investigate it, more studies are necessary to prove the efficacy and security in the long-term.

## CONFLICT OF INTEREST STATEMENT

The authors declare they have no conflicts of interest to disclose.
